# Health and wellness in the Australian coal mining industry: An analysis of pre-post findings from the RESHAPE workplace health promotion program

**DOI:** 10.1371/journal.pone.0288244

**Published:** 2023-07-07

**Authors:** Aaron Bezzina B., Lee Ashton, Trent Watson, Carole L. James

**Affiliations:** 1 Centre for Resources Health and Safety, College of Health, Medicine and Wellbeing, University of Newcastle, Callaghan, Australia; 2 School of Health Sciences, College of Health, Medicine and Wellbeing, University of Newcastle, Callaghan, Australia; 3 Active Living Research Program, Hunter Medical Research Institute (HMRI), New Lambton Heights, New South Wales, Australia; 4 School of Education, College of Human and Social Futures, University of Newcastle, Callaghan, Australia; 5 Ethos Health, Newcastle, Australia; 6 Sydney School of Health Sciences, Faculty of Medicine and Health, The University of Sydney, Camperdown, New South Wales, Australia; Christian Medical College, INDIA

## Abstract

**Objectives:**

Non-communicable diseases are the leading cause of death worldwide, accounting for 71% of deaths in 2021. The chronic and pervasive nature of these diseases spurs the need for novel treatment approaches, including using the workplace as a front for the promotion and dissemination of health messages and activities. Taking this into consideration, this study aimed to assess the efficacy of a workplace health promotion program that targeted nutrition, physical activity, and obesity outcomes in a New South Wales (NSW) coal mine site.

**Design:**

A 12-week quasi-experimental pre-test–post-test study.

**Setting:**

A coal mine site in rural NSW, Australia.

**Participants:**

At baseline there were n = 389 participants, with similar numbers at follow-up (n = 420) as well as 61 participants from both timepoints (8.2% repeated measures), with 89% of participants being male.

**Intervention:**

A multicomponent wellness intervention was implemented incorporating aspects of education, goal setting, and competition.

**Outcomes:**

Physical activity, nutrition, and weight.

**Results:**

The mean BMI at baseline was 30.01 kg/m^2^, and at follow-up 29.79 kg/m^2^ (p = 0.39). At follow-up, participants reported 81% lower odds of engaging in the exercise category ‘no moderate physical exercise’ (OR = 0.09, p < 0.001), as well as 111% higher odds of meeting physical activity and exercise guidelines (OR = 2.11, p = 0.057). There were no changes to diet outcomes and no association between employment characteristics and participating in physical activity.

**Conclusions:**

Workplace health promotion programs can be an efficacious strategy in improving physical activity outcomes and marginally improving weight outcomes in those in the mining industry. Further research is needed to determine the true effectiveness of these programs long-term, particularly in an environment as challenging and dynamic as the mining industry.

## Background

Noncommunicable diseases (NCDs) are the leading causes of death and disability worldwide [1]. Reports from the World Health Organization estimate that in 2021, 71% of deaths globally were attributable to NCDs, rising from 63% in 2013 [2, 3]. Lifestyle diseases like cardiovascular disease (CVD), type 2 diabetes and obesity continue to be a treatment conundrum for public health policy makers. The ever-ubiquitous nature of overweight and obesity [4], alongside the rising prevalence of type 2 diabetes compounds treatment approaches that aim to reduce the debilitating effects of CVD [1, 5]. Evidently, disability-adjusted life years for CVD have risen by 16.4% between the years 2007 and 2017 [6], further underscoring the need for action.

Considering the pervasive nature of these diseases, novel treatment approaches have been proposed, including using the workplace as a front for the dissemination and promotion of positive health behaviors [7]. Modifiable health behaviors such as physical inactivity, poor diet, excessive alcohol consumption and tobacco smoking, can all negatively contribute to NCDs [8]. Hence, health programs which look to target these behaviors in the workplace could prove valuable in the ongoing fight to curb the morbidity and mortality of these diseases.

Workplace health promotion programs offer a coordinated and strategic approach to health promotion, empowering employees to live healthier lives through policies, environmental support, and health promotion activities which can be conducted in the workplace [9]. There is a growing evidence base to support these programs [7], especially with regards to modifying dietary and obesogenic habits, as well as improving cardiometabolic health outcomes [7]. Specifically, a meta-analysis of 121 studies by Peñalvo et al., [7], found that workplace wellness programs can statistically improve fruit and vegetable consumption (0.27 servings per day [95% CI 0.16 to 0.37]), as well as systolic blood pressure (–2.03 mm Hg [–3.16 to –0.89]) and bodyweight (–0.92 kg [–1.11 to –0.72]) [7]. Other studies have investigated effects of workplace physical activity interventions on men, and have found mixed success [10].

As a front for health promotion, workplaces offer the necessary infrastructure and sustained reach to a large number of individuals for prolonged periods each day [11]. This larger reach also means workplaces have access to demographics who would otherwise be difficult to engage in health promotion. Blue-collar male dominated industries such as mining [12], construction [13], and manufacturing [14], could all potentially benefit greatly from workplace health programs as this population is usually underrepresented in health research [15], yet may benefit the most from such interventions [16].

Globally, Australia is one of the biggest mining nations [17], and is just one example of a blue-collar male dominated industry that could benefit from workplace health promotion. The Minerals Council of Australia, the country’s governing mining industry body reaffirms that core to the industry, there is a strong emphasis on, and commitment to the safety, health, and psychological wellbeing of its workforce [18]. Despite this, the physical health and wellbeing of employees in the industry is notably falling behind [12, 19, 20]. The industry comprises of approximately 90% males [12], with roughly 85% of males in the industry classified as overweight or obese [21]. Employees in this industry have also reported lower consumption of fruit and vegetables compared to gender and aged match general population samples [12, 22]. In a cross section of 949 coal miners across New South Wales (NSW), Australia, it has been reported that only 3.5% of employees meet current recommendations for vegetable intake, as well as 42.2% for fruit (compared to 9% and 51.4% nationally) [12]. This is in conjunction with an episodic hazardous drinking culture [23], capped off by an obesogenic work environment whereby employees spend large parts of the day sedentary.

Knowledge gaps remain about the success of diet and physical activity programs within blue-collar workers, particularly miners. This paper presents baseline and follow-up findings from a pilot study that implemented a 12-week multicomponent workplace health promotion program at a mine site in rural NSW, Australia. The study aimed to assess the efficacy of a workplace health program that targeted nutrition, physical activity, and obesity outcomes, implemented under the broader RESHAPE framework. Secondary outcomes of interest were whether workplace factors contributed to poorer health behaviors.

## Materials and methods

RESHAPE is an eight-step framework based on the WHO ‘Health Workplace Framework and Model’ [24]. The framework was developed in response to an industry identified need that was highlighted from the *‘Blueprint for the management of overweight and obesity in the NSW mining industry’* [25]. Coal mining, and mining more generally, is an incredibly dynamic and variable environment. As such, RESHAPE does not adopt the mantra of one size fits all, rather it looks at problems pragmatically, and applies appropriate mine site specific solutions. Whilst this study is focusing on the issue of overweight and obesity within the workplace, the RESHAPE process is designed to be used for any modifiable health risk factor including smoking, nutrition, alcohol, physical activity, sleep or mental health.

The aim of RESHAPE is to provide a sustained approach to fostering healthy, happy, safe, and productive workplaces. Through shared responsibility, the workplace can become an environment where healthy choices are valued. This produces organizational change through ongoing investment into workplace health and cultivates a workplace culture which is conducive to positive change. RESHAPE is a systematic approach to workplace wellness. Encompassed within this framework are wellness programs which this paper looks to discuss.

### Ethics

The project was approved by the University of Newcastle Human Research Ethics Committee (approval number H-2019-0087).

### Study design

This study used a quasi-experimental pre-test–post-test design, whereby the study participants across the one mine site were measured at baseline and again 19 months later.

### Recruitment

Three NSW mine sites, two open cut and one underground, were recruited to participate in the pilot study via a convenience sample of mine sites that expressed interest in participation. Sites that expressed initial interest (n = 6) were sent an information package (PowerPoint slideshow and digital implementation booklet) detailing the RESHAPE process and philosophy. Three sites joined the study, from the Hunter and Central West regions of NSW, Australia. The study was capped at three sites for funding reasons and a combination of underground and open cut was elected to capture different workplace characteristics. Due to the COVID-19 global pandemic, two study sites withdrew participation from the study citing operational issues brought about by the disease. In between lockdowns, one site was able to complete follow-up data. This paper reports pre-post data on the one site that was able to complete the full intervention.

### Intervention

The wellness intervention took a whole site approach whereby participation was open to all employees on site. All employees were made aware of the intervention and were encouraged to participate; however, the final level of involvement is unknown. The intervention was a commercially available 12-week multicomponent wellness program which was paid for by grant funding and made freely available to all employees. The program comprised of twelve weekly health focuses, with an emphasis on nutrition and physical activity to drive changes to obesity outcomes. The intervention used several aspects of gender targeting to increase intervention uptake across the site, including: [1] healthy weight challenge; [2] step challenge (who could do the most steps); [3] Core stability challenge (time plank adjusted for age) and [4] Healthy recipe mini challenge (submission of best home cooked recipes that was scored against a criterion for originality, presentation, and nutrition value).

The program included aspects of behavior change techniques including goal setting, self-monitoring (through an online website), modification of eating habits and problem-solving, and a large emphasis on knowledge and education. Goal setting was conducted via a generalized template asking several questions including: “Why is your health important to you?”, “What aspects of your lifestyle could you improve” and, “How could you improve these lifestyle behaviors?”. Participants were encouraged to set three action orientated goals. In conjunction with the nutrition aspects of the program, employees were educated about the Australian physical activity and exercise guidelines [22], and encouraged to increase physical activity to these levels. Handouts and communications were delivered to participating employees’ email, and participants logged into an online portal to record information about their diet and exercise. Table 1 outlines additional intervention aspects and flow.

**Table 1:** 12-week intervention breakdown.

Week	Handouts^a^	Touch points	Challenges
**1**	Launch	F2F workshops, Inbody screening	Healthy weight challenge (BW, % body fat)
**2**	Energy Balance	Pre-start PP slides, electronic handout	
**3**	Resistance training & increasing exercise intensity	Pre-start PP slides, electronic handout	Step challenge
**4**	Portion control	Pre-start PP slides, electronic handout	
**5**	Core stability	Pre-start PP slides, electronic handout	Core stability mini challenge
**6**	Nutrition labelling	Pre-start PP slides, electronic handout	
**7**	Sport/Exercise nutrition		
**8**	Healthy eating out		
**9**	Heart health	Pre-start PP slides, electronic handout	
**10**	Eating and fatigue		
**11**	Meal prep and smart shopping		Healthy recipe mine challenge
**12**	Prizes^b^	Inbody screening	Healthy weight challenge (BW, % body fat)

Notes:

^a^ Handouts were in the form of 1–4 pages booklets containing information about the focus of the week. During the first week of the program, the face-to-face workshop was conducted by a dietitian at the monthly training days, for a duration of 1 hour. The whole workforce attended the workshop. The pre-start talks were delivered by the front-line leader or Health and Safety coordinator at their pre-start meeting prior to shift.

^b^ Prizes were for overall winners was used to promote health and fitness alongside some friendly competition. The Grand Prize which was given to the individual who lost the highest percentage body weight and included a weekend away valued at AUD$1000. The major prize for the highest body fat percentage lost was a AUD$500 voucher. For the mini challenges, 1st place received a AUD$100 voucher, 2nd place: AUD$75, voucher, and 3rd place: AUD$50 voucher.

### Wellness surveys

Paper based baseline surveys were conducted in August 2019, with follow-up surveys completed in February 2021 during staff training days. Originally, follow-up data collection was scheduled for August 2020, 12 months after baseline. However, due to the COVID-19 pandemic, this was not feasible, and the alternative date was chosen.

An information statement attached to the survey explained the aims of the project, the voluntary nature of the research, and the confidentiality of data collected. To link responses from baseline to follow-up, participants were asked to enter their surname and year of birth to generate a unique study code. Consent was obtained via a tick box at the end of the information statement. The expected completion time of the survey was 15–20 minutes.

The survey assessed a range of outcomes including personal characteristics which were a mix of closed and open-ended questions. Personal characteristics gauged aspects concerned with the participants highest education, relationship status, and language spoken at home. Occupation aspects were also assessed and examined the participants employment status, shift work status, hours worked per week, and their employment role. Occupational metrics were all closed questions apart from the question on hours worked per week which was open ended.

The main portion of the wellness survey was centered around validated and reliable health surveying instruments. These included the Epworth Sleepiness Scale, an eight-item closed ended instrument for assessing sleep and daytime sleepiness [26]. The Generalized Anxiety Disorder 2-item (GAD-2) and the Patient Health Questionnaire-2 (PHQ-2) which look to screen for generalized anxiety disorder and depressive disorder respectively [27, 28]. The Fagerstrom test for nicotine dependence is a six-item instrument which assess smoking outcomes [29, 30]. and the short form Alcohol Use Disorders Identification Test (AUDIT-C) which is a three-item instrument to determine alcohol dependency, and drinking outcomes [31, 32]. Lastly, questions regarding nutrition intake and physical activity were all adapted from the 2017–2018 Australian National Health Survey [22], and were validated for use in a general Australian population [33]. Nutrition questions concerned with fruit and vegetable intake, sugar sweetened beverage consumption and fast-food occasions were all closed questions with examples regarding serving sizes provided. Physical activity and exercise questions as per the Australian National Health Survey were a mix of open and closed questions gauging participants moderate and vigorous physical activity as well as walking and resistance training [22].

### Statistical analysis

Descriptive statistics were created to summarize the demographic information and outcome measures at baseline and follow-up. Categorical variables were summarized through frequencies and percentages (n (%)). Numerical variables were summarized through mean and standard deviation (Mean (SD)), and median and interquartile range (Median (Q1, Q3)). Statistical analyses were programmed using SAS v9.4 (SAS Institute, Cary, North Carolina, USA).

### Primary analysis

Differences in outcomes between baseline and follow-up were compared using mixed modelling, with a random intercept to account for the repeated measures on participants. Not all participants had repeated measures, and it was impossible to discern whether participants with missing IDs had repeated measures. All participants with missing IDs were considered unique participants with no repeated measures for the purposes of the analysis.

Each model was then adjusted for the potential confounder (the confounder was found by comparing differences in proportion for demographic/work related information between time points). Various models appropriate to each outcome were used:(I)Mixed normal: Body Mass Index (BMI), Weight; (II)Mixed logistic: Physical activity and exercise guidelines; (III) Mixed ordinal: Generalized Anxiety Disorder 2-item (GAD-2) score, Patient Health Questionnaire-2 (PHQ-2) score, Alcohol Use Disorders Identification Test Consumption (AUDIT-C) score, Servings of fruits/vegetables/sugar sweetened beverages/fast food consumed per day; (IV) Mixed zero-inflated gamma: Minutes of moderate physical exercise per week, Minutes of vigorous physical exercise per week.

### Secondary analysis

The association between the outcomes and predictors was modelled in the same manner detailed for the primary analysis.

### Sensitivity analysis

Due to missing data on the outcomes, sensitivity analyses were also performed on each outcome, imputing missing data via multiple imputation by chained equations (MICE) under the missing at random (MAR) assumption. The association between missing data on each outcome and demographic/work-related characteristics (age, gender, highest civilian qualification, relationship status, occupation, employment status, usual hours worked per week, shift work status) were utilized to inform the imputation models. For both the primary analysis and secondary analysis, effect estimates were stable after n = 50 imputed data sets (Table 2).

**Table 2:** Sensitivity results health related outcome (m = 50).

		Crude			Adjusted^a^		
Outcome	Statistic	Estimate 95% CI)	P-value	Obs	Estimate (95% CI)	P-value	Obs
**BMI**	LS Means: Baseline	29.91 (29.36, 30.45)	-	29.99 (29.36, 30.61)	-	29.91 (29.36, 30.45)	-
	LS Means: Follow-up	29.86 (29.36, 30.36)	-	29.97 (29.32, 30.61)	-	29.86 (29.36, 30.36)	-
	LS Mean Difference: Baseline to Follow-up	-0.04 (-0.73, 0.65)	0.904	-0.02 (-0.72, 0.68)	0.959	-0.04 (-0.73, 0.65)	0.904
**Weight (kg)**	LS Means: Baseline	93.55 (91.84, 95.25)	-	94.25 (92.27, 96.24)	-	93.55 (91.84, 95.25)	-
	LS Means: Follow-up	93.27 (91.74, 94.79)	-	94.08 (92.12, 96.04)	-	93.27 (91.74, 94.79)	-
	LS Mean Difference: Baseline to Follow-up	-0.28 (-2.44, 1.88)	0.798	-0.17 (-2.36, 2.02)	0.878	-0.28 (-2.44, 1.88)	0.798
**Physical activity and exercise guidelines**	Odds Ratio	1.75 (1.00, 3.06)	0.051	1.76 (1.00, 3.09)	0.049	1.75 (1.00, 3.06)	0.051
**Minutes of Moderate Physical Exercise Per Week**	Logistic: Odds Ratio	0.10 (0.05, 0.20)	< .001	0.10 (0.05, 0.20)	< .001	0.10 (0.05, 0.20)	< .001
	Gamma: Rate Ratio	1.06 (0.92, 1.23)	0.415	1.05 (0.91, 1.22)	0.490	1.06 (0.92, 1.23)	0.415
**Minutes of Vigorous Physical Exercise Per Week**	Logistic: Odds Ratio	0.53 (0.35, 0.82)	0.004	0.55 (0.36, 0.84)	0.006	0.53 (0.35, 0.82)	0.004
	Gamma: Rate Ratio	0.93 (0.78, 1.12)	0.455	0.94 (0.79, 1.13)	0.537	0.93 (0.78, 1.12)	0.455
**Fruit serves per day**	Odds Ratio	1.15 (0.74, 1.78)	0.524	1.11 (0.71, 1.74)	0.638	1.15 (0.74, 1.78)	0.524
**Vegetable serves per day**	Odds Ratio	0.91 (0.62, 1.33)	0.617	0.89 (0.60, 1.31)	0.549	0.91 (0.62, 1.33)	0.617
**Sugar sweetened beverages per day**	Odds Ratio	1.24 (0.81, 1.89)	0.323	1.23 (0.80, 1.90)	0.350	1.24 (0.81, 1.89)	0.323
**Fast food consumed**	Odds Ratio	1.04 (0.72, 1.51)	0.830	1.09 (0.74, 1.59)	0.659	1.04 (0.72, 1.51)	0.830
**GAD-2 Score**	Odds Ratio	0.70 (0.44, 1.10)	0.119	0.65 (0.40, 1.05)	0.077	0.70 (0.44, 1.10)	0.119
**PHQ-2 Score**	Odds Ratio	1.24 (0.87, 1.76)	0.245	1.15 (0.79, 1.67)	0.459	1.24 (0.87, 1.76)	0.245
**AUDIT-C Score**	Odds Ratio	0.98 (0.65, 1.47)	0.907	1.02 (0.67, 1.56)	0.924	0.98 (0.65, 1.47)	0.907

^a^Adjusted for usual hours worked per week.

Abbreviations: Body Mass Index (BMI), Generalized Anxiety Disorder 2-item (GAD-2), Patient Health Questionnaire-2 (PHQ-2), Alcohol Use Disorders Identification Test Consumption (AUDIT-C)

## Results

There were n = 389 records at baseline and n = 420 recorded at follow-up, as well as 61 participants from both timepoints (8.2% repeated measures) (Table 3). Surveys were distributed to all employees onsite with a total of 556 surveys distributed at baseline and 583 at follow-up. This represents a response rate of 70% and 72% respectively at each timepoint. At baseline and follow-up, majority of participants belonged to the 25–34 age bracket or 35–44 age bracket (32% and 31% respectively). Most of the participants were male (89%) and were born in Australia (93%).

**Table 3:** Participant demographic characteristics.

Characteristics	Response	Baseline (n = 389)	Follow-up (n = 420)	Total (n = 809)	P-value
**Age**	18–24	11 (3.6%)	28 (6.7%)	39 (5.4%)	0.487
	25–34	101 (33%)	132 (32%)	233 (32%)	0.487
	35–44	96 (31%)	125 (30%)	221 (31%)	0.487
	45–54	70 (23%)	92 (22%)	162 (22%)	0.487
	55–74	29 (9.4%)	40 (9.6%)	69 (9.5%)	0.487
	Missing	82	3	85	0.487
**Gender**	Male	345 (90%)	363 (88%)	708 (89%)	0.451
	Female	36 (9.4%)	40 (9.7%)	76 (9.5%)	0.451
	Other	2 (0.5%)	4 (1.0%)	6 (0.8%)	0.451
	Prefer not to say	1 (0.3%)	5 (1.2%)	6 (0.8%)	0.451
	Missing	5	8	13	0.451
**Highest qualification**	No formal qualifications/Prefer not to say	15 (3.9%)	19 (4.6%)	34 (4.3%)	0.171
	School certificate/Higher school certificate	145 (38%)	127 (31%)	272 (34%)	0.171
	University Degree/Higher University Degree	23 (6.0%)	24 (5.8%)	47 (5.9%)	0.171
	Trade/apprenticeship	134 (35%)	151 (36%)	285 (36%)	0.171
	Certificate/Diploma	65 (17%)	93 (22%)	158 (20%)	0.171
	Missing	7	6	13	0.171
**Country born**	Australia	358 (93%)	391 (93%)	749 (93%)	0.783
	Other	28 (7.3%)	28 (6.7%)	56 (7.0%)	0.783
	Missing	3	1	4	0.783
**Language spoken at home**	English	381 (99.2%)	413 (99%)	794 (99%)	0.509
	Another language	3 (0.8%)	6 (1.4%)	9 (1.1%)	0.509
	Missing	5	1	6	0.509
**Relationship status**	Single	58 (15%)	66 (16%)	124 (15%)	0.309
	Widowed/Divorced/ Separated/In a relationship but not living together	31 (8.1%)	28 (6.7%)	59 (7.4%)	0.309
	Married/Defacto/ Living together	291 (76%)	318 (76%)	609 (76%)	0.309
	Prefer not to say	2 (0.5%)	8 (1.9%)	10 (1.2%)	0.309
	Missing	7	0	7	0.309
**Employment Status**	Permanent full-time employment	311 (82%)	316 (75%)	627 (78%)	0.053
	Part-time employment	21 (5.5%)	24 (5.7%)	45 (5.6%)	0.053
	Other	49 (13%)	80 (19%)	129 (16%)	0.053
	Missing	8	0	8	0.053
**Usual hours worked per week**	0–38 hours	73 (20%)	38 (9.7%)	111 (15%)	<0.001
	39–45 hours	175 (47%)	231 (59%)	406 (53%)	<0.001
	46–56 hours	95 (26%)	107 (27%)	202 (27%)	<0.001
	56+ hours	27 (7.3%)	16 (4.1%)	43 (5.6%)	<0.001
	Missing	19	28	47	<0.001
**Shift work status**	Yes	310 (81%)	343 (82%)	653 (82%)	0.784
	No	72 (19%)	75 (18%)	147 (18%)	0.784
	Missing	7	2	9	0.784

The highest qualification obtained varied amongst participants with a trade / apprenticeship (36%) being the most reported, followed by school certificate / higher school certificate (34%). Most participants were married or in a de facto (living together but not married) relationship (76%) and permanent full-time employees (78%). Hours worked per week varied with 39–45 hours being the most reported (53%), followed by 46–56 hours (27%), as well as most employees participating in shift work (82%).

Table 4 shows summary statistics for the outcomes. Numeric outcomes are summarized through mean (SD) and median (Q1, Q3), and categorical outcomes are summarized through frequencies and percentages (n (%)). The mean BMI at baseline was 30.01 kg/m^2^, and at follow-up 29.79 kg/m^2^. The percentage of participants not meeting the Australian physical activity and exercise guidelines decreased between baseline and follow-up (61% to 52% respectfully), in addition to a mean increase in the number of moderate physical activity minutes participated in per week between baseline and follow-up (153 minutes to 201 minutes). Dietary consumption patterns are reported across the varying cut offs. At baseline and follow-up, the most reported fruit intake was 1 serve per day, and similarly for vegetables, 1 serve per day.

**Table 4:** Summary statistics of assessed outcome variables.

Outcome	Response	Baseline (n = 389)	Follow-up (n = 420)	Total (n = 809)
**BMI**	Mean (SD)	30.01 (5.17)	29.79 (5.28)	29.89 (5.23)
	Median (Q1, Q3)	29.30 (26.58, 32.94)	29.17 (26.12, 32.54)	29.30 (26.26, 32.66)
**Weight (kg)**	Mean (SD)	94 (17)	93 (16)	94 (16)
	Median (Q1, Q3)	92 (82, 105)	92 (82, 105)	92 (82, 105)
**Physical activity and exercise guidelines**	Not meeting guideline	172 (61%)	133 (52%)	305 (57%)
	Meeting guideline	109 (39%)	124 (48%)	233 (43%)
**Minutes of Moderate Physical Exercise Per Week**	Mean (SD)	153 (255)	201 (178)	176 (223)
	Median (Q1, Q3)	90 (0, 210)	123 (60, 300)	120 (45, 240)
**Minutes of Vigorous Physical**				
**Exercise Per Week**	Mean (SD)	109 (205)	112 (141)	110 (177)
	Median (Q1, Q3)	30 (0, 120)	60 (0, 180)	60 (0, 150)
**Fruit serves per day**	I never eat fruit	17 (4.5%)	23 (5.7%)	40 (5.1%)
	Less than 1 serve a day	85 (22%)	81 (20%)	166 (21%)
	1 serve per day	130 (34%)	157 (39%)	287 (37%)
	2 serve per day	97 (26%)	96 (24%)	193 (25%)
	3 serve per day	36 (9.5%)	31 (7.7%)	67 (8.6%)
	4 serve per day	7 (1.9%)	11 (2.7%)	18 (2.3%)
	5 serve per day	5 (1.3%)	5 (1.2%)	10 (1.3%)
	6 or more serves per day	1 (0.3%)	1 (0.2%)	2 (0.3%)
**Vegetable serves per day**	I never eat vegetables	4 (1.1%)	1 (0.2%)	5 (0.6%)
	Less than 1 serve a day	31 (8.2%)	41 (10%)	72 (9.2%)
	1 serve per day	145 (38%)	135 (33%)	280 (36%)
	2 serve per day	108 (28%)	129 (32%)	237 (30%)
	3 serve per day	50 (13%)	59 (14%)	109 (14%)
	4 serve per day	29 (7.7%)	28 (6.9%)	57 (7.3%)
	5 serve per day	9 (2.4%)	8 (2.0%)	17 (2.2%)
	6 or more serves per day	3 (0.8%)	6 (1.5%)	9 (1.1%)
**Sugar Sweetened Beverages per day**	I never drink soft drink, cordial, flavored water, or sport drinks	33 (8.7%)	32 (7.9%)	65 (8.3%)
	Less than 1 cup per month	38 (10%)	44 (11%)	82 (10%)
	1 cup per week	70 (18%)	78 (19%)	148 (19%)
	2–6 cups per week	87 (23%)	98 (24%)	185 (24%)
	1 cup per day	53 (14%)	62 (15%)	115 (15%)
	2 cups per day	38 (10%)	44 (11%)	82 (10%)
	3 cups per day	31 (8.2%)	28 (6.9%)	59 (7.5%)
	4 cups per day	15 (3.9%)	9 (2.2%)	24 (3.1%)
	5 cups per day	5 (1.3%)	3 (0.7%)	8 (1.0%)
	6 cups per day	5 (1.3%)	2 (0.5%)	7 (0.9%)
	7 cups per day	0	1 (0.2%)	1 (0.1%)
	8 cups per day	1 (0.3%)	0	1 (0.1%)
	More than 8 cups per day	4 (1.1%)	3 (0.7%)	7 (0.9%)
**Fast food consumed**	Never	13 (3.4%)	13 (3.2%)	26 (3.3%)
	Less than 1 per month	79 (21%)	67 (16%)	146 (19%)
	1–3 times per wee	59 (16%)	121 (30%)	180 (23%)
	Once per week	153 (40%)	116 (29%)	269 (34%)
	2 times per week	39 (10%)	62 (15%)	101 (13%)
	3 times per week	16 (4.2%)	16 (3.9%)	32 (4.1%)
	4 times per week	10 (2.6%)	5 (1.2%)	15 (1.9%)
	5 times per week	6 (1.6%)	1 (0.2%)	7 (0.9%)
	6 times per week	0	1 (0.2%)	1 (0.1%)
	7 times per week	3 (0.8%)	2 (0.5%)	5 (0.6%)
	More than 7 times per week	0	3 (0.7%)	3 (0.4%)
**GAD-2 Score**	Mean (SD)	1 (1)	1 (1)	1 (1)
	Median (Q1, Q3)	0 (0, 1)	0 (0, 1)	0 (0, 1)
**PHQ-2 Score**	Mean (SD)	1 (1)	1 (1)	1 (1)
	Median (Q1, Q3)	0 (0, 1)	0 (0, 1)	0 (0, 1)
**AUDIT-C Score**	Mean (SD)	5 (3)	6 (3)	5 (3)
	Median (Q1, Q3)	6 (3, 8)	5 (4, 8)	6 (4, 8)

Abbreviations: Body Mass Index (BMI), Generalized Anxiety Disorder 2-item (GAD-2), Patient Health Questionnaire-2 (PHQ-2), Alcohol Use Disorders Identification Test Consumption (AUDIT-C)

Table 5 shows the crude and adjusted estimates for each outcome. Odds ratios and rate ratios are follow-up when compared to baseline. For BMI, in the crude models, the mean BMI at baseline was 30.02 and the mean BMI at follow-up was 29.76. The difference between baseline and follow-up is -0.27, but this was not statistically significant (p = 0.39) and similarly for the adjusted model -0.22 (p = 0.52). From baseline to follow-up, the crude model demonstrated that there were no statistically significant changes regarding fruit intake (OR = 1.13, p = 0.58), vegetable intake (OR = 0.93, p = 0.73), sugar sweetened beverages (OR = 1.29, p = 0.26), and fast-food intake (OR = 1.03, p = 0.86).

**Table 5:** Crude and adjusted estimates for each outcome comparing follow-up against baseline.

Outcome		Crude			Adjusted^a^		
	Statistic	Estimate (95% CI)	P-value	Obs	Estimate (95% CI)	P-value	Obs
**BMI**	LS Means: Baseline	30.02 (29.48, 30.57)	-	695	30.01 (29.37, 30.66)	-	659
	LS Means: Follow-up	29.76 (29.26, 30.25)	-	695	29.79 (29.14, 30.45)	-	659
	LS Mean Difference: Baseline to Follow-up	-0.27 (-0.89, 0.35)	0.389	695	-0.22 (-0.91, 0.47)	0.520	659
**Weight (kg)**	LS Means: Baseline	93.92 (92.33, 95.50)	-	733	94.61 (92.68, 96.54)	-	694
	LS Means: Follow-up	93.04 (91.58, 94.50)	-	733	93.71 (91.76, 95.67)	-	694
	LS Mean Difference: Baseline to Follow-up	-0.88 (-2.65, 0.90)	0.327	733	-0.89 (-2.86, 1.08)	0.364	694
**Physical activity and exercise guidelines**	Odds Ratio	2.11 (0.98, 4.56)	0.057	538	2.37 (1.05, 5.35)	0.039	508
**Minutes of Moderate Physical Exercise Per Week**	Logistic: Odds Ratio	0.09 (0.04, 0.20)	< .001	991	0.10 (0.05, 0.22)	< .001	937
	Gamma: Rate Ratio	1.10 (0.94, 1.28)	0.253	991	1.10 (0.93, 1.31)	0.236	937
**Minutes of Vigorous Physical Exercise Per Week**	Logistic: Odds Ratio	0.46 (0.26, 0.81)	0.008	860	0.49 (0.28, 0.86)	0.014	819
	Gamma: Rate Ratio	0.98 (0.80, 1.20)	0.867	860	1.00 (0.81, 1.24)	0.967	819
**Fruit serves per day**	Odds Ratio	1.13 (0.72, 1.78)	0.580	783	1.24 (0.75, 2.03)	0.394	740
**Vegetable serves per day**	Odds Ratio	0.93 (0.63, 1.39)	0.727	786	0.82 (0.52, 1.29)	0.391	742
**Sugar sweetened beverages per day**	Odds Ratio	1.29 (0.82, 2.01)	0.263	784	1.36 (0.85, 2.19)	0.198	741
**Fast food consumed**	Odds Ratio	1.03 (0.71, 1.51)	0.864	785	1.09 (0.73, 1.63)	0.650	742
**GAD-2 Score**	Odds Ratio	0.72 (0.46, 1.15)	0.168	785	0.61 (0.36, 1.05)	0.073	739
**PHQ-2 Score**	Odds Ratio	1.25 (0.86, 1.81)	0.243	793	1.05 (0.69, 1.60)	0.808	747
**AUDIT-C Score**	Odds Ratio	1.00 (0.61, 1.62)	0.992	764	1.04 (0.61, 1.78)	0.891	720

^a^Adjusted for usual hours worked per week.

Abbreviations: Body Mass Index (BMI), Generalized Anxiety Disorder 2-item (GAD-2), Patient Health Questionnaire-2 (PHQ-2), Alcohol Use Disorders Identification Test Consumption (AUDIT-C)

For physical activity and exercise guidelines in the crude models, people at follow-up had 111% higher odds of meeting the physical activity and exercise guidelines compared to people at baseline, but this was not statistically significant (OR = 2.11, p = 0.057). For moderate physical exercise in the crude models, people at follow-up had 81% lower odds of doing no moderate physical exercise compared to people at baseline, and this was statistically significant (OR = 0.09, p < 0.001). Furthermore, people at follow-up had 10% increase in the number of minutes spent doing moderate physical exercise when compared to people at baseline, but this was not statistically significant (RR = 1.10, p = 0.236).

Table 6 examined workplace factors as predictors of exercise outcomes. Physical activity and exercise guidelines, Minutes of moderate physical exercise per week, and Minutes of vigorous physical exercise per week were examined against the factors of interest. For Physical activity and exercise guidelines, people who were employed part-time had 5% higher odds of meeting the guidelines compared to people employed full-time, but this was not statistically significant (OR = 1.05, p = 0.96).

**Table 6:** Modelling of exercise related outcomes. OR = Odds Ratio, RR = Rate Ratio.

		Exercise and physical activity guidelines		Moderate Activity				Vigorous Activity			
		Binary		Binary		Gamma		Binary		Gamma	
Predictor	Response	OR (95% CI)	P-value	OR (95% CI)	P-value	OR (95% CI)	P-value	OR (95% CI)	P-value	OR (95% CI)	P-value
**Timepoint**	Baseline	Reference	-	Reference	-	Reference	-	Reference	-	Reference	-
	Follow-up	2.08 (0.85, 5.06)	0.102	0.12 (0.05, 0.26)	< .001	1.06 (0.90, 1.27)	0.467	0.53 (0.29, 0.96)	0.037	1.05 (0.84, 1.31)	0.639
**Shift work status**	Yes	Reference	-	Reference	-	Reference	-	Reference	-	Reference	-
	No	2.05 (0.55, 7.59)	0.264	1.26 (0.54, 2.96)	0.587	1.17 (0.92, 1.48)	0.186	0.79 (0.36, 1.69)	0.522	1.08 (0.80, 1.45)	0.618
**Employment**	Permanent full-time	Reference	-	Reference	-	Reference	-	Reference	-	Reference	-
	Other	2.40 (0.66, 8.70)	0.171	1.49 (0.68, 3.27)	0.305	1.14 (0.90, 1.44)	0.265	0.81 (0.39, 1.65)	0.544	1.04 (0.77, 1.42)	0.769
	Part-time employment	1.05 (0.16, 7.00)	0.960	0.28 (0.03, 2.62)	0.255	0.99 (0.68, 1.44)	0.970	0.83 (0.27, 2.60)	0.744	1.44 (0.88, 2.35)	0.138
**Usual hours per week**	0–38 hours	Reference	-	Reference	-	Reference	-	Reference	-	Reference	-
	39–45 hours	0.67 (0.17, 2.63)	0.544	0.79 (0.35, 1.79)	0.564	1.12 (0.85, 1.47)	0.403	0.61 (0.28, 1.36)	0.214	0.72 (0.49, 1.06)	0.092
	46–56 hours	0.93 (0.21, 4.03)	0.916	0.85 (0.34, 2.13)	0.722	1.03 (0.77, 1.40)	0.817	0.50 (0.20, 1.21)	0.116	0.80 (0.53, 1.20)	0.272
	56+ hours	0.87 (0.10, 7.22)	0.892	0.68 (0.18, 2.62)	0.563	0.94 (0.61, 1.44)	0.760	0.83 (0.25, 2.83)	0.762	0.67 (0.37, 1.22)	0.180
**Occupation**	Machinery operator	Reference	-	Reference	-	Reference	-	Reference	-	Reference	-
	Manger	2.23 (0.25, 19.91)	0.450	0.37 (0.04, 3.58)	0.380	0.91 (0.60, 1.37)	0.637	0.52 (0.13, 2.07)	0.338	0.90 (0.54, 1.52)	0.691
	Professional / Administration/ Other	1.01 (0.09, 11.84)	0.994	0.93 (0.19, 4.51)	0.923	0.74 (0.45, 1.21)	0.215	0.36 (0.07, 1.94)	0.224	0.79 (0.44, 1.42)	0.422
	Trade worker	1.66 (0.59, 4.64)	0.312	0.87 (0.44, 1.73)	0.688	1.18 (0.97, 1.43)	0.091	0.67 (0.36, 1.25)	0.196	0.98 (0.76, 1.27)	0.891
**Relationship status**	Single	Reference	-	Reference	-	Reference	-	Reference	-	Reference	-
	Married/Defacto/ Living together	1.28 (0.21, 7.68)	0.774	1.15 (0.32, 4.17)	0.829	0.78 (0.55, 1.11)	0.157	1.52 (0.51, 4.51)	0.438	1.18 (0.74, 1.89)	0.471
	Widowed/Divorced/ Separated	0.57 (0.17, 1.89)	0.336	1.31 (0.57, 3.04)	0.513	0.78 (0.62, 0.98)	0.031	1.43 (0.69, 2.95)	0.320	0.97 (0.72, 1.29)	0.807

For moderate activity (binary), people at follow-up had 97% lower odds of not doing any moderate activity compared to people at baseline, and this was statistically significant (OR = 0.03, p < 0.001). For vigorous activity (gamma), managers had 10% lower number of minutes spent performing vigorous activity compared to machinery operators, but this was not statistically significant (RR = 0.9, p = 0.690).

## Discussion

This study underscores the potential benefits of workplace health programs in improving employee’s modifiable health behaviors within the coal mining industry. Noting that people at follow-up compared to baseline had 111% (p = 0.057) higher odds of meeting physical activity and exercise guidelines, and 81% lower odds of doing no moderate physical exercise (OR = 0.09, p < 0.001). When examining predictors of exercise outcomes against workplace factors (shift work status, employment type, hours worked per week), there were not significant associations. Notably, there was no differences in fruit (p = 0.39) and vegetable (p = 0.39) intake between timepoints.

Physical inactivity has established links with a raft of non-communicable diseases and premature mortality [34, 35]. So much so, that globally, 7.6% of cardiovascular disease deaths can be attributed to physical inactivity, and similarly for all-causes of death (7.2%) [36]. Combined with the notion that one-in-four adults globally do not meet current physical activity and exercise guidelines [37], there is significant potential for workplace health promotion programs that target these behaviors. Considering the efficacious results of this paper, as well as others [7, 38], the authors posit that the workplace should be more heavily utilized in the promotion of physical activity and associated health behaviors. One particular study which found similar success in increasing physical activity outcomes in a similar male dominated industry was the Preventing Obesity Without Eating like a Rabbit (POWER) study by Morgan et al., [39]. Amongst 110 workers with overweight or obesity in a manufacturing plant in Australia, during a 14 week wait-list randomized control trial, workers in the intervention group significantly increased current physical activity levels (p<0.001). Similarities between the POWER study and this study was the utilization of behavior change techniques (BCTs) in intervention materials and design which has been highlighted as a powerful tool for workplace health programs that target modifiable health behaviors in men and male dominated industries [38].

Behavior change techniques can be thought of as the active ingredients of behavior change interventions [40]. Meta-analytic models by Sharp et al., [41], have shown in males, the utilization of BCTs is a key driver in increasing physical activity in intervention studies. Overall, behavior change interventions can have a small but significant effect on increasing physical activity levels (d = 0.35) in men [41]. This effect size is consistent with an increase of approximately 97 minutes of total physical activity per week or 980 steps per day [41]. Interestingly, studies that employed four or more BCTs were found to have a larger effect size (d = 0.51; 95% CI 0.38,0.63) than studies that used three or less (d = 0.24; 95% CI 0.15,0.34) [41]. Utilizing appropriate BCTs to increase participation and uptake of physical activity intervention could prove an advantageous design decision for workplace health programs to targeting modifiable health behaviors [34, 35].

Workplace health programs have shown effectiveness in statistically reducing overall body weight. Peñalvo et al., [7], recently conducted a systematic review and meta-analysis assessing the effectiveness of workplace wellness programs for dietary habits, overweight, and cardiometabolic health. The review included 121 studies and found that workplace wellness programs significantly reduced BMI (–0.22 kg/m2 [95% CI –0.28 to –0.17]), bodyweight (–0.92 kg [–1.11 to –0.72]), and waist circumference (–1.47 cm [–1.96 to –0.98]. Whilst the findings from this study could not replicate the statistical significance produced by the aforementioned review, differences of -0.88kg body weight (p = 0.32) draws attention to the potential of these programs in improving health outcomes [7].

Considering this, from a health and safety perspective, even modest changes to weight can have substantial impacts on safety outcomes within the mining industry and the workplace more broadly. Wilson [42], reinforces this notion in a study investigating safety incident risk of 868 underground and opencut miners over a 5-year period. The study found that for every one-unit increase BMI over a reference 25 (upper threshold of healthy), there was an 8% (p < 0.05) increase in incident risk. Additionally, Maisey et al., [43], when studying workplace fatigue in fly-in-fly-out mining workers found that for every 1 unit increase in BMI, the odds of risk for obstructive sleep apnea increased by 19% (p < 0.05). Obstructive sleep apnea is one of the most prevalent sleep disorders the mining population (31%), second only to shift work disorder (44%), and a significant contributor to fatigue [43]. Safety issues such as fatigue pose a threat to worker health and safety with an estimated 65% of truck driving accidents in open cut mines being fatigue related [44]. The benefits of workplace health programs can extend beyond the individual behaviors they are targeting and double down as a safety incident risk management measure.

Strengths of this study include a large sample size and an intervention that took a whole of site approach. This approach has several benefits as implementing a health program in a non-controlled environment allows findings to be more applicable to industry, thus increasing the translation of research into practice. Limitations include not knowing the exact level of participation in the wellness intervention. Whilst all employees were made aware of the intervention and could participate, the level of involvement is unknown. Additionally, due to COVID-19 extending the follow-up timepoint (due to site access constraints), potential contamination regarding wellness outcomes ought to be acknowledged. Furthermore, this study acknowledges the limitation of not including data on pre-existing medical conditions, which could have provided additional context regarding the importance of workplace health promotion programs. Lastly, reporting bias due to the self-reported nature of measures and the small sample of paired data should be recognized. Due to the small sample of paired data, caution is prescribed in interpreting results on an individual level. Despite this, due to the high response rate at baseline and follow-up (70% and 72% respectively), the authors believe these results still offer a snapshot of the workplace as a whole.

## Conclusions

This study assessed the efficacy of a multicomponent workplace health program targeting modifiable health behaviors in NSW coal miners. Notably, improvements to physical activity outcomes and to a lesser degree, marginal weight loss does indicate that the workplace could be a viable option in health promotion and improving employee’s health outcomes. Further research is needed to determine the true effectiveness of these programs, particularly in an environment as challenging and dynamic as the mining industry.
