# Correction: Health and wellness in the Australian coal mining industry: An analysis of pre-post findings from the RESHAPE workplace health promotion program

**DOI:** 10.1371/journal.pone.0319223

**Published:** 2025-02-10

**Authors:** Aaron Bezzina, Lee Ashton, Trent Watson, Carole L. James

The first author’s name is spelled incorrectly. The correct name is: Aaron Bezzina. The correct citation is: Bezzina A, Ashton L, Watson T, James CL (2023) Health and wellness in the Australian coal mining industry: An analysis of pre-post findings from the RESHAPE workplace health promotion program. PLoS ONE 18(7): e0288244. https://doi.org/10.1371/journal.pone.0288244

Table 2 also includes incorrect headings. The correct Table 2 is provided here.

**Table 2:** Sensitivity results health related outcome (m = 50).

		Crude		Adjusted*	
Outcome	Statistic	Estimate (95% CI)	P-value	Estimate (95% CI)	P-value
BMI	LS Means: Baseline	29.91 (29.36, 30.45)	-	29.99 (29.36, 30.61)	-
	LS Means: Follow-up	29.86 (29.36, 30.36)	-	29.97 (29.32, 30.61)	-
	LS Mean Difference: Baseline to Follow-up	-0.04 (-0.73, 0.65)	0.904	-0.02 (-0.72, 0.68)	0.959
Weight (kg)	LS Means: Baseline	93.55 (91.84, 95.25)	-	94.25 (92.27, 96.24)	-
	LS Means: Follow-up	93.27 (91.74, 94.79)	-	94.08 (92.12, 96.04)	-
	LS Mean Difference: Baseline to Follow-up	-0.28 (-2.44, 1.88)	0.798	-0.17 (-2.36, 2.02)	0.878
Guideline	Odds Ratio	1.75 (1.00, 3.06)	0.051	1.76 (1.00, 3.09)	0.049
Minutes of Moderate Physical Exercise Per Week	Logistic: Odds Ratio	0.10 (0.05, 0.20)	< .001	0.10 (0.05, 0.20)	< .001
	Gamma: Rate Ratio	1.06 (0.92, 1.23)	0.415	1.05 (0.91, 1.22)	0.490
Minutes of Vigorous Physical Exercise Per Week	Logistic: Odds Ratio	0.53 (0.35, 0.82)	0.004	0.55 (0.36, 0.84)	0.006
	Gamma: Rate Ratio	0.93 (0.78, 1.12)	0.455	0.94 (0.79, 1.13)	0.537
Fruit serves per day	Odds Ratio	1.15 (0.74, 1.78)	0.524	1.11 (0.71, 1.74)	0.638
Vegetable serves per day	Odds Ratio	0.91 (0.62, 1.33)	0.617	0.89 (0.60, 1.31)	0.549
Sugar Sweetened Beverages per day	Odds Ratio	1.24 (0.81, 1.89)	0.323	1.23 (0.80, 1.90)	0.350
Fast food consumed	Odds Ratio	1.04 (0.72, 1.51)	0.830	1.09 (0.74, 1.59)	0.659
GAD-2 Score	Odds Ratio	0.70 (0.44, 1.10)	0.119	0.65 (0.40, 1.05)	0.077
PHQ-2 Score	Odds Ratio	1.24 (0.87, 1.76)	0.245	1.15 (0.79, 1.67)	0.459
AUDIT-C Score	Odds Ratio	0.98 (0.65, 1.47)	0.907	1.02 (0.67, 1.56)	0.924

*adjusted for usual hours worked per week.
